# The rubber hand illusion questionnaire: An exploratory graph analysis of ownership, referral of touch, and control statements

**DOI:** 10.3758/s13414-024-02964-w

**Published:** 2024-09-30

**Authors:** Giorgia Tosi, Andreas Kalckert, Anantha Krishna Sivasubramanian, Daniele Romano

**Affiliations:** 1 Department of Psychology, University of Milano-Bicocca, Milano, Italy; 2 Department of Cognitive Neuroscience and Philosophy, University of Skövde, 54128 Skövde, Sweden; 3 School of Psychology and Clinical Language Sciences, University of Reading Malaysia, Iskandar Puteri, Malaysia

**Keywords:** Rubber hand illusion, Exploratory graph analysis, Questionnaire, Sense of ownership, Body awareness, Psychometric

## Abstract

The rubber hand illusion is a well-known experiment that allows manipulation of one's own body experience. The original questionnaire gauges both the illusion experience and unforeseen experiences acting as control statements. In particular, two statements pertain to the referral of touch originating from the rubber hand (RoT), and one concerns the feeling of ownership of the rubber hand (SoO). Despite its prominence, the rubber hand illusion questionnaire has not undergone thorough examination of its psychometric properties. The literature reveals a tendency to use RoT and SoO statements interchangeably. In this study, we employed Exploratory Graph Analysis to explore the item structure and compared the correlation between SoO and RoT items in various conditions. While SoO and RoT are closely linked, our findings suggest potential separation. SoO and RoT statements consistently correlate, yet hints emerge that RoT might represent a distinct facet of the illusion. Correlations diminish beyond the perihand space, indicating a nuanced relationship. Additionally, moderate relationships between control statements and those measuring the illusion suggest that even control statements are modulated by the illusion experience. This study underscores the need for further exploration into the psychometric properties of body illusion questionnaires, prompting reflections on the interpretation in light of these results.

## Introduction

The rubber hand illusion (RHI) is a well-known experiment that allows researchers to manipulate the experience of the own body. In this experiment, participants perceive a fake model to be part of their own body (= sense of body ownership, SoO). Introduced in 1998 by Botvinick and Cohen, it has gained much attention, with hundreds of studies emerging since its first publication. Meanwhile, a number of variants have been introduced, for example a full-body illusion (Petkova & Ehrsson, 2008), an enfacement illusion (Tsakiris, 2008), and a moving rubber hand (Kalckert & Ehrsson, 2012). These experiments increased our understanding of the perceptual and cognitive processes involved in the experience of the own body (Ehrsson, 2011; Kilteni et al., 2015), and have further demonstrated subsequent effects of body ownership illusions on cognitive processes (see e.g., Pyasik et al., 2022; Tosi & Romano, 2023).

Body ownership illusions are typically assessed by three different approaches: questionnaires, the proprioceptive drift, and skin conductance response after a simulated threat. The former approach uses statements reflecting the illusion experience, and participants rate these statements in relation to their subjective experience during different conditions. The latter two reflect a behavioral dimension of the illusion. In the proprioceptive drift task, participants either point to or visually estimate the location of the hand that has been affected by the illusion (Riemer et al., 2013; Tsakiris et al., 2006). Typically, these tasks show that participants locate their hand closer to the position of the rubber hand during the illusion, as compared to non-illusion-related conditions. Although the validity of this task has been questioned by some studies (Holle et al., 2011; Rohde et al., 2011), a recent meta-analysis has shown that the proprioceptive drift task shows a positive correlation to the subjective illusion experience (Tosi et al., 2023). The skin conductance response measurement simulates a threat to the rubber hand with a knife or syringe, and measures the physiological response to this threat (Armel & Ramachandran, 2003; Ehrsson et al., 2007). Here, typically an elevated response can be measured during illusion conditions as compared to non-illusion conditions.

The first study of the RHI by Botvinick and Cohen (1998) introduced two of these three approaches (i.e., questionnaire and proprioceptive drift), and they remain widely used in the field. Many studies up to this day use the original questionnaire as introduced by this study. Botvinick and Cohen (1998) used nine statements that were rated on a 7-point Likert scale. The first three were illusion statements reflecting the intended experience of the illusion, whereas the other six statements were considered unrelated (= unpredicted) experiences that can be considered as control statements. These statements are thought to control for compliance or desirability effects, and are typically negatively rated (i.e., are denied by participants during the RHI paradigm). Typically, an RHI questionnaire consists of such illusion and control statements, with different formulations and/or a different number of statements.

The original questionnaire contained three illusion related statements: (1) “It seemed as if I were feeling the touch of the paintbrush in the location where I saw the rubber hand touch”; (2) “It seemed as though the touch I felt was caused by the paintbrush touching the rubber hand”; and (3) “I felt as if the rubber hand were my hand” (Botvinick & Cohen, 1998). Although these statements have been consistently rated highly during illusion-conditions and found to be key components (see, e.g., Longo et al., 2008), they do represent different aspects within the illusion experience: statements 1 and 2 are statements that reflect the experience of the touch that seems to originate from the rubber hand during the illusion (referral of touch, RoT). It can be understood as a visual capture of the tactile sensation on the participant's hand towards the visual stimulus applied on the rubber hand (Pavani et al., 2000; Rock & Victor, 1964). Statement 3 instead reflects the experience of ownership of the rubber hand, i.e., the experience that the rubber hand is part of my own body or *my* hand (SoO). Both these facets often co-appear and are correlated, thus are seemingly contingent on each other (Makin et al., 2008).

The RHI questionnaire and its statements have not been extensively examined in terms of its psychometric properties. Longo et al. (2008) used a principal component approach of 27 statements that reflected different dimensions of the illusion experiences. This analysis identified four different main components of the illusion: embodiment of the rubber hand, loss of the own hand, movement, and affect. The embodiment of the rubber hand in turn was structured out of three different aspects: the feeling of perceiving the rubber hand as part of the body (Sense of Ownership, SoO), the feeling of co-localizing the sensation of the two hands (relocation of the hand, and referral of touch), and the expectation to be able to move the rubber hand (Sense of Agency, SoA) (Longo et al., 2008). Interestingly, the RoT statement (see statement 8, Table 1; Longo et al., 2008) had one of the lowest loadings within this component. Recently, Romano et al. (2021) examined a larger cohort of participants with the RHI paradigm administering the same 27-statement questionnaire. The Principal Component Analysis (PCA) on the experimental condition identified three components: the embodiment of the rubber hand, the disembodiment of the own hand, and physical sensations. The authors recovered the subcomponents of the embodiment sensation (i.e., SoO, RoT, SoA) only as a suboptimal solution (Romano et al., 2021). Tosi and Romano (2023) extended these to the full-body illusion, and used Exploratory Graph Analysis (EGA). EGA is a recent method developed in the context of network analysis to estimate the number of communities underlying a set of correlated variables (Golino et al., 2019, 2020; Golino & Epskamp, 2017). The authors identified four communities: SoO, SoA, Sense of Co-location (concerning the location of the fake limbs and the disappearance of the real ones), and Disembodiment of the own body. Crucially, the item assessing RoT did not show any connection with the other items. These studies show a lack of agreement about the relationship between SoO and RoT.

**Table 1:** Matrix of Exploratory Graph Analysis (EGA) network loadings in the synchronous condition. The table shows statement loadings for each factor

Item	Question	1	3	2
O1	I felt as if the rubber hand was part of my body	**0.402**	0.237	0.085
O2	I felt as if the rubber hand was my hand	**0.396**	0.101	0.170
R2	I felt as if the touch was caused by the brush touching the rubber hand	**0.388**	0.093	0.004
R1	I felt the touch of the brush in the location where I saw the rubber hand being touched/I felt the touch of the paintbrush on the rubber hand	**0.287**	0.061	0.167
C1	I felt as if my real hand was turning rubbery	0.151	**0.288**	0.160
C2	I felt as if I had no longer a right hand, as if my right hand had disappeared	0.153	**0.288**	0.033
C3	It seemed as if the touch I was feeling came from somewhere between my own right hand and the rubber hand	0.170	0.030	**0.266**
C4	It seems as if I had more than one right hand	0.073	0.148	**0.266**

When inspecting the literature, it becomes apparent that these two statement categories are often used interchangeably. There is a large heterogeneity in questionnaire approaches: some studies may use SoO statements, some RoT, some a mix of these, and most studies do not a priori define how the ratings of these different statements specifically reflect successful RHI induction (Kalckert, Bico et al., 2019a). Some studies analyze the data by producing an aggregate score by summing all illusion-related statements (i.e., an illusion index; see e.g., Abdulkarim & Ehrsson, 2016), others use the individual ratings only. Subsequently, the scores are compared either against the same scores of a control condition or against the control statements. These approaches may cover or uncover certain trends of the illusion experience as reflected in the ratings of the respective statement categories, depending on the analytical approach and conceptualization of the illusion.

Kalckert, Bico et al. (2019a), examined an RHI data set of 40 participants and analyzed both these aspects of SoO versus RoT. In this experiment, it has been shown that both these statement categories can be rated differently. Specifically, it has been shown that when a balloon, i.e., a non-bodily object, is used instead of a rubber hand, the RoT statements are seemingly more affirmed (i.e., a positive rating of > 0), whereas SoO statements are denied (i.e., a negative rating of < -1). When producing an aggregate score by averaging the ratings of both statement categories though, this difference between these statement categories may be obscured. Further, it has been shown that the number of responders (i.e., participants who affirm the presence of the respective illusion aspect) differs, with more individuals affirming the RoT, giving it higher ratings (see also Reader et al., 2021). Taken together, these studies raise the question of how SoO and RoT are related and how these are rated in specific manipulations. These observations emphasize the need for a better understanding of the RHI questionnaire.

In the current study, we re-examine pooled data from different experiments (Kalckert, Perera et al., 2019b; Sivasubramaniam, 2023). Crucially, by pooling the data, this allows analytical approaches such as EGA (Tosi & Romano, 2023), which is widely used in psychometric studies for questionnaire data but requires a relatively large sample size (Epskamp, 2016). Most RHI studies typically operate with around 20 participants only. The present data are using a questionnaire with a limited number of statements, unlike larger psychometric studies. It represents a typically used RHI questionnaire akin to the original one by Botvinick and Cohen using a set number of different statements, but also with an equal number of statements for SoO and RoT. EGA allows us to analyze questionnaire data from two perspectives. It provides an item-level look into the correlations between the items and highlights the unique association between any two variables after conditioning on all others. Moreover, this technique allows the identification of items’ communities, which are conceptualized as groups of variables more correlated between them than with the rest of the network. EGA allows us to understand how items gather around a common latent variable (i.e., community) without losing the item-level correlation analysis. This cannot be done with a regular PCA, and it is therefore suitable for our scope. The present work's primary aim was indeed to explore the item-level structure of the RHI questionnaire, still considering the overarching latent structure. Specifically, we were interested in the relationship between SoO and RoT items.

## Methods

### Participants

The present data come from six different experiments: Experiments 1 and 2 of Kalckert, Perera, et al. (2019b) (n = 99), and four experiments by Sivasubramanian (2023) (Exp.1, n = 46; Exp. 2, n = 22; Exp. 3, n = 17; Exp. 4, n = 72), collected during the period 2018–2022. The total number of participants is 212. For our purpose, we use only the data from the synchronous and asynchronous trials that are comparable across these experiments (EGA, total sample size N = 195, 130 female, mean age and standard deviation 22.84 ± 6.46 years; age range = 18–44 years) and repeat this for data from trials in which the distance is increased (correlation comparison, total sample size N = 144, 93 female, mean age and standard deviation = 23.46 ± 6.55 years; age range = 18–44 years). The participants gave written informed consent. All experiments were approved by the University of Reading Malaysia ethics committee. None of the studies were pre-registered.

### Apparatus and stimuli

Within these experiments, participants underwent trials with synchronous stimulation, in which the brush strokes to the rubber and the real hand are carried out simultaneously, plus additional manipulations such as asynchronous stimulation (i.e., in which the brush strokes are delayed approximately 500 ms) or different distances between the two hands (i.e., near vs. far space). Each condition lasted 90 s and participants received brush strokes at a 1-Hz pace, with a limited number of pseudo-random double strokes. Touches were delivered to the proximal phalanx of the index finger with a light brush. For the participants included in the EGA, the two hands were placed laterally to each other, and the distance was 13 cm (Kalckert, Perera et al., 2019b) or 15 cm, respectively (Sivasubramaniam, 2023). For the participants included in the correlation comparison, we used the data from Experiment 1 of Kalckert, Perera et al. (2019b) and Experiments 2 and 3 of Sivasubramanian (2023) in which the distance between the two hands was increased to 38 cm or 45 cm in the far condition, respectively. For more details see the respective publications. In both instances (near and far distance), the distance varied minimally across the different experiments. These distances are either clearly within limits of the perihand space (< 15 cm) that allow the illusion to occur (Kalckert & Ehrsson, 2014) and are used in most RHI studies (Riemer et al., 2019), or beyond the perihand space (> 35 cm) (Serino et al., 2015) that abolishes the illusion (Kalckert & Ehrsson, 2014; Preston, 2013). Kalckert, Perera et al. (2019b) used a manual stimulation procedure, and Sivasubramanian (2023) used an automated stimulation protocol with a custom-built stimulation device (Sivasubramaniam et al., 2021). It should be noted though that the frequency and characteristics of the touch were highly similar across these experiments, as the stimulation delivery by the device by Sivasubramanian et al. (2021) mimicked the manual procedure of Kalckert, Perera et al. (2019b). Further, it should be noted that no differences between the manual versus automated stimulation protocol have been found using this device and protocol (Sivasubramaniam et al., 2021). Thus, we consider the data to be generated under comparable experimental circumstances.

### Questionnaire

The present data used eight statements, which represent a typically used RHI questionnaire. In particular, the questionnaire contained four illusion-related statements reflecting SoO (items O1 and O2) and RoT (items R1 and R2), and four control statements (items C1, C2, C3, and C4). The full list of the items is reported in (Table 1). Participants rated their agreement with the statements on a 7-point Likert scale (from -3: total disagreement, to +3: total agreement, zero indicated uncertainty).

### Analysis

The analyses were performed using the EGAnet (Version 1.2.3; Christensen & Golino, 2021) and cocor (Version 1.1-4; Diedenhofen & Musch, 2015) packages of the R statistical software (Version 4.3.0; R Core Team, 2017).

#### Exploratory graph analysis (*EGA*)

EGA applies the Gaussian Graphical Model (GGM) to estimate the network from a group of variables, followed by a community detection algorithm. A network is defined by a set of nodes (i.e., the variables of interest) and edges connecting those nodes (i.e., their relationships). In GGM, edges are calculated as regularized partial correlation coefficients and give the relationship between two variables after conditioning on all other variables in the dataset (Epskamp & Fried, 2018). GGM uses the “least absolute shrinkage and selection operator” (LASSO) algorithm (Tibshirani, 2011) to reduce spurious correlation. A tuning parameter lambda (λ) controls for the density of the network (i.e., the presence of non-0-value correlations; Epskamp, 2016). Lower values of λ increase the possibility of including spurious correlations, while larger values of λ grow the probability of removing relevant edges. In the present study, the minimum and maximum λ ratio was set to 0.01. The choice of the best tuning parameter is based on the extended Bayesian Information Criterion (eBIC). The eBIC applies a hyperparameter gamma (γ), which controls how much it prefers simple models with fewer edges (Foygel & Drton, 2010). Larger γ values lead to simpler models, and smaller γ values lead to denser models. In the present study, we set γ to 0.5, as suggested by the literature (Epskamp, 2016). After the network selection, EGA uses the walktrap algorithm (Pons & Latapy, 2006) to find the number of communities. The algorithm calculates a transition matrix based on the sum of the partialized correlation between each node. Each matrix value represents the probability of one node traversing to another. Random walks are then initiated for a certain number of steps (that we set to four) using the transition matrix for probable destinations. Each node starts as its own community, which merges with adjacent communities to minimize the sum of squared distances between communities (i.e., Ward’s agglomerative clustering approach; Ward, 1963). The optimal partition of communities is determined through modularity (Newman, 2006), such as a node’s community is determined by its proportion of many densely connected edges to few sparsely connected edges (Christensen & Golino, 2021).

After performing EGA, we calculated the network sparsity (i.e., the proportion of 0-value correlations on the total number of possible correlations), the network mean weight (i.e., the average edges weight in absolute value), and the strength of the nodes (i.e., a centrality index computed as the sum of all absolute edge weights connected to a given node). We checked for the dimension stability of EGA results through a bootstrap analysis (Christensen & Golino, 2021). To this end, we generated 10,000 networks by resampling from the original data with replacement (i.e., maintaining the same number of cases as the original data). EGA was then applied to the replicated data, resulting in a sampling distribution of EGA networks. From this sampling distribution, we obtained the likelihood of dimensions (i.e., the distribution of the proportion of times that a certain number of dimensions was replicated), the items’ stability (i.e., the replication rate of each item in the empirical dimensions), and the average loadings across bootstrap.

We ran the analyses on (i) the synchronous condition items and (ii) the delta score computed as the difference between synchronous and asynchronous conditions. The delta score is often computed to assess the illusion effect net of the general effect induced by the experimental paradigm. We aimed to explore the item-level structure of the embodiment questionnaire in different conditions. Specifically, we were interested in the relationship between SoO and RoT items.

#### Correlation comparison

We used the method implemented by Zou (2007) to compare two non-overlapping correlations based on dependent groups. The test calculates the confidence interval of the difference between two correlations. If the confidence interval includes zero, the null hypothesis that the two correlations are equal must be retained. If the confidence interval does not include zero, the null hypothesis has to be rejected (Zou, 2007).

We considered the data from synchronous stimulation in two conditions: when the rubber hand was placed within the limits of the perihand space (< 15 cm) or beyond the perihand space (> 35 cm). For each condition (within vs. beyond), we computed the average scores for SoO and RoT items, respectively. Then, we assessed whether the correlation between SoO and RoT within the perihand space differs from that beyond the perihand space.

The data and analysis code are available via the Open Science Framework (OSF) at the following link: https://osf.io/3687e/?view_only=11995653f88b4a3cb0ca65d116555051

## Results

**Fig. 1:**
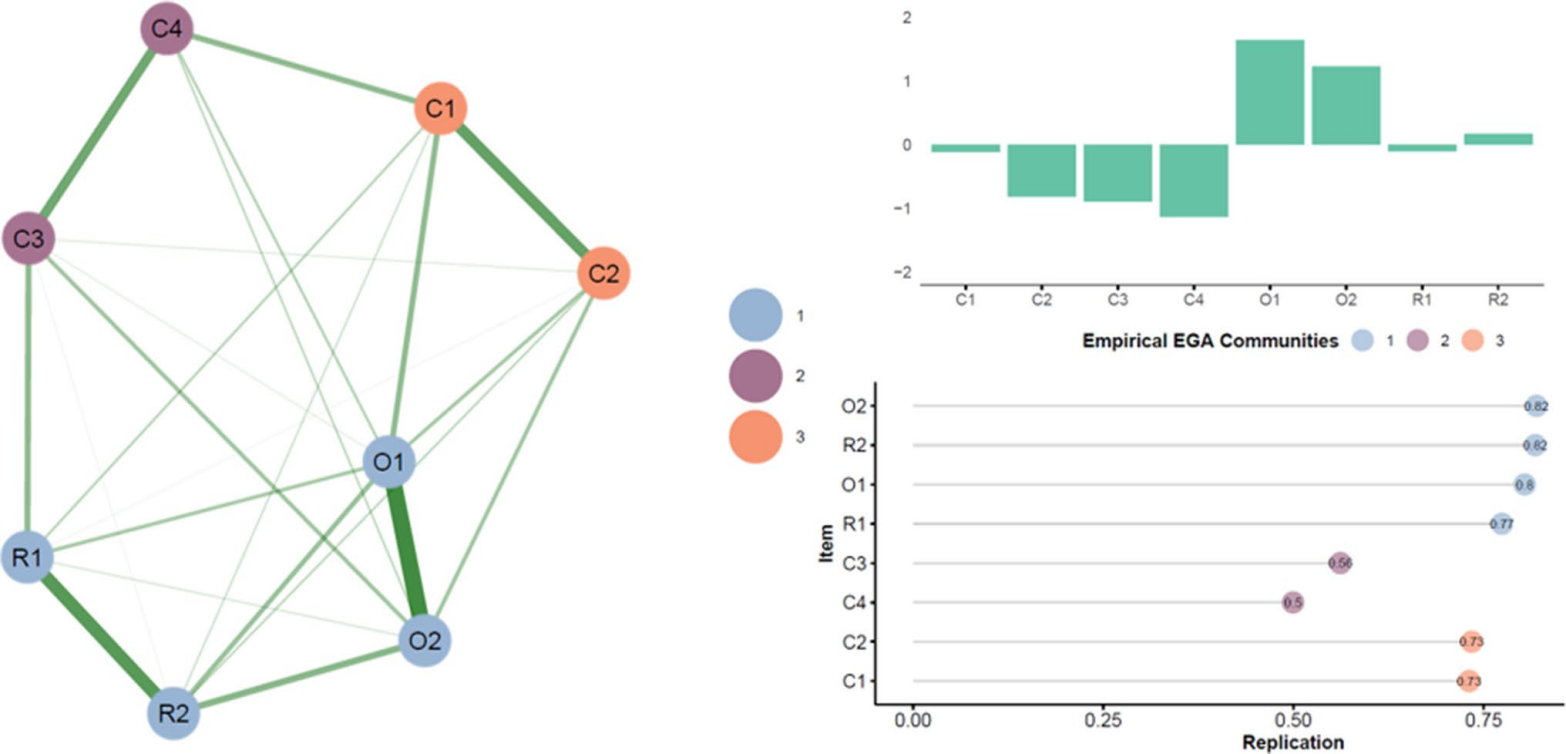
EGA results in the synchronous condition. Panel (**a**) shows the best network estimated by the Exploratory Graph Analysis. The edges represent regularized partial correlations. Green lines indicate positive associations. Red lines would have indicated negative associations. The edges’ size and color saturation represent the relationships’ intensity. The nodes indicate the items in the questionnaire colored by their community. Panel (**b**) shows the standardized node strength. Panel (**c**) shows the nodes replication rates in empirical communities

The synchronous condition network comprehended eight nodes connected by 23 edges (density: 0.821). The mean weight was 0.124, suggesting relatively low correlations (the exact weights of all edges and the simple correlations among all variables are reported in Table 2, together with means and standard deviations). The items related to the SoO showed the highest strength (Fig. 1, panel b), suggesting their centrality in the network. EGA revealed three communities (Fig. 1, panel a). The first community captured the items related to SoO and RoT (items O1, O2, R1, and R2), the second community comprehended control items related to supernumerary limbs (items C3 and C4), and the third community was formed by control items related to disownership sensation (items C1 and C2). Table 1 reports the communities’ loading for each item.

**Table 2:** Simple correlations and network weights in the synchronous condition. The lower triangle reports simple correlations, measured with Pearson’s correlation. The upper triangle reports the network weights, which correspond to regularized partial correlations. The diagonal reports the mean and the standard deviation of each item

	O1	O2	R1	R2	C1	C2	C3	C4
**O1**	0.595 ± 1.999	0.547	0.114	0.150	0.182	0.113	0.018	0.080
**O2**	0.847	0.590 ± 1.957	0.042	0.210	0.000	0.126	0.128	0.068
**R1**	0.659	0.644	1.164 ± 1.890	0.423	0.072	0.004	0.193	0.000
**R2**	0.718	0.720	0.730	0.738 ± 1.880	0.050	0.066	0.005	0.000
**C1**	0.651	0.582	0.528	0.557	-0.180 ± 1.901	0.359	0.000	0.185
**C2**	0.631	0.616	0.496	0.549	0.649	-0.713 ± 1.902	0.038	0.000
**C3**	0.530	0.552	0.530	0.487	0.431	0.421	0.056 ± 1.845	0.307
**C4**	0.523	0.516	0.401	0.436	0.500	0.402	0.539	-0.508 ± 1.911

The bootstrap procedure revealed that most of the items had a good replication rate in the empirical communities (Fig. 1, panel c). However, items C3 and C4 showed low stability with similar replication rates in the other communities. Moreover, the three-community structure (likelihood: 0.409) and the two-community structure (likelihood: 0.475) had similar replication frequencies. Although the solution with four communities was suboptimal (likelihood: 0.116), the averaged loadings showed that when a fourth community is recovered, it is loaded by the RoT items (Table 3). Also, the RoT items had, on average, higher loadings on the fourth community than on the empirical one (i.e., first community).

**Table 3:** Averaged loadings across bootstraps in the synchronous condition. The table shows the average statements loadings considering all bootstrap replicas

	Community 1	Community 2	Community 3	Community 4
**O2**	0.363	0.171	0.172	0.172
**R2**	0.328	0.086	0.115	0.345
**O1**	0.370	0.119	0.254	0.188
**R1**	0.278	0.167	0.091	0.345
**C3**	0.160	0.284	0.112	0.126
**C4**	0.111	0.274	0.186	0.007
**C2**	0.164	0.048	0.292	0.049
**C1**	0.170	0.141	0.327	0.090

**Fig. 2:**
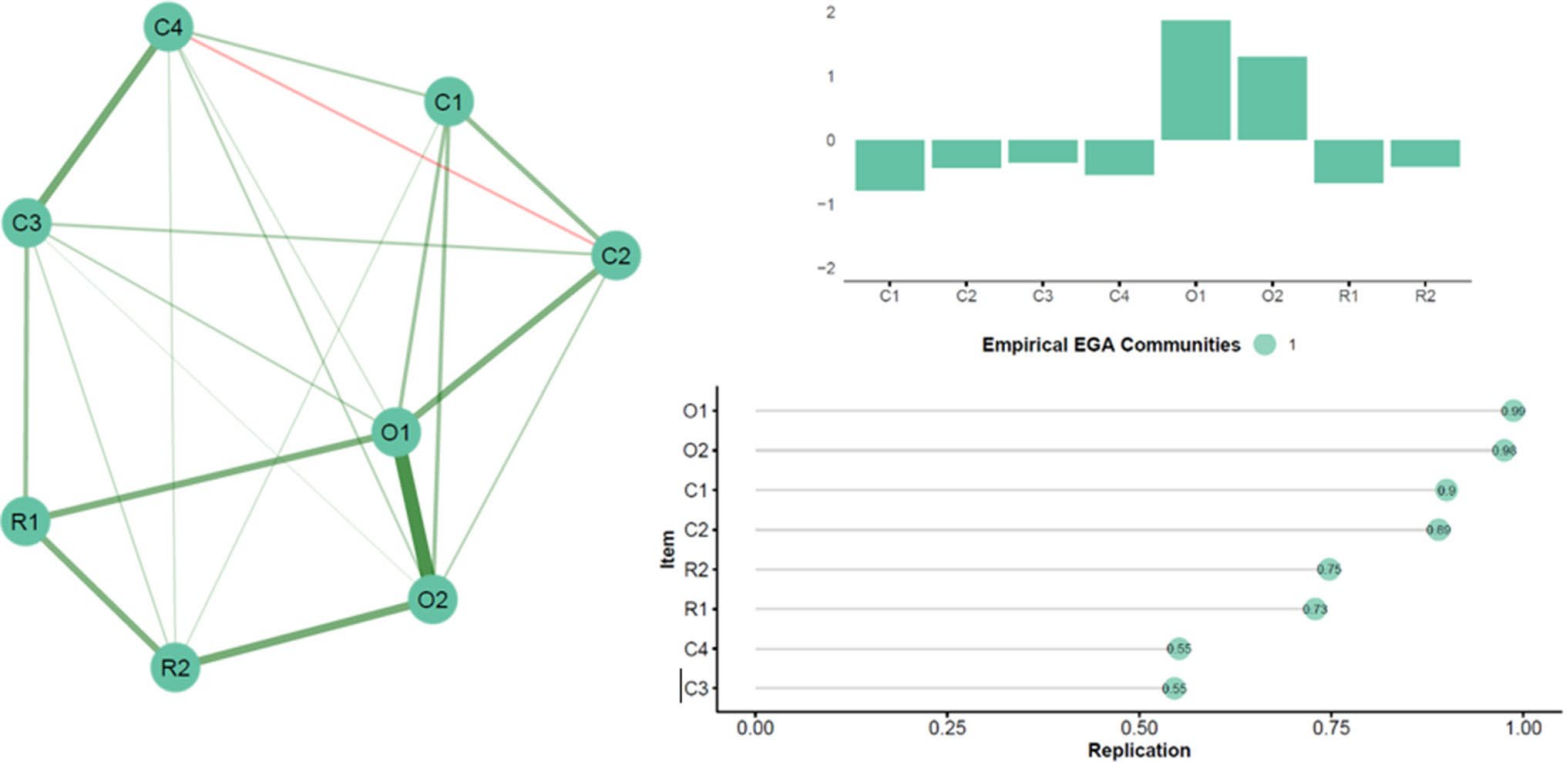
Exploratory Graph Analysis (EGA) results in the delta condition. The left panel shows the best network estimated by the EGA. The edges represent regularized partial correlations. Green lines indicate positive associations. Red lines would have indicated negative associations. The size and the color saturation of the edges represent the intensity of the relationships. The nodes indicate the items in the questionnaire colored by the community they belong to. The right panel shows the standardized node strength and the nodes replication rates in empirical communities

To access the item-level structure net of the general effect induced by the experimental paradigm, we conducted the same analysis on a delta score computed as the difference between synchronous and asynchronous conditions. The delta network comprehended eight nodes connected by 21 edges (density: 0.750). The mean weight was 0.112, confirming the presence of relatively low correlations (the exact weights of all edges and the simple correlations among all variables are reported in Table 4). Again, the item related to the SoO showed the highest strength (Fig. 2, panel b). EGA revealed one community (Fig. 2, panel a), suggesting that the variation between conditions is unidimensional. Again, items C3 and C4 showed low stability with similar replication rates in other communities (Fig. 2, panel c).

**Table 4:** Simple correlations and network weights in the delta condition. The lower triangle reports simple correlations, measured with Pearson’s. The upper triangle reports the network weights, which correspond to regularized partial correlations. The diagonal reports the mean and the standard deviation

	O1	O2	R1	R2	C1	C2	C3	C4
**O1**	1.400 ± 1.829	0.485	0.240	0.000	0.135	0.240	0.076	0.048
**O2**	0.7084923	1.400 ± 1.972	0.000	0.281	0.142	0.081	0.025	0.078
**R1**	0.478	0.397	1.477 ± 2.034	0.233	0.000	0.000	0.157	0.000
**R2**	0.421	0.511	0.424	1.487 ± 1.887	0.053	0.000	0.065	0.057
**C1**	0.453	0.448	0.232	0.298	0.621 ± 1.566	0.171	0.000	0.101
**C2**	0.490	0.418	0.214	0.214	0.371	0.631 ± 1.611	0.098	-0.094
**C3**	0.364	0.333	0.345	0.296	0.206	0.261	0.441 ± 1.653	0.282
**C4**	0.283	0.298	0.192	0.248	0.244	0.072	0.385	0.446 ± 1.903

Surprisingly, the bootstrap results revealed that the two-community solution was the most frequently replicated (likelihood: 0.849) as compared to the other solutions (unidimensional likelihood: 0.000; three-community likelihood: 0.148, four-community likelihood: 0.003). The average loadings across bootstrap (Table 5) showed similar loadings between dimensions, making it challenging to define which items were related to the second community.

**Table 5:** Averaged loadings across bootstraps in the delta condition

	Community 1	Community 2	Community 3	Community 4
**O1**	0.462	0.198	0.122	0.156
**O2**	0.422	0.165	0.097	0.070
**R1**	0.232	0100	0.089	0.065
**R2**	0.251	0.087	0.053	0.046
**C1**	0.246	0.172	0.080	0.060
**C2**	0.224	0.186	0.118	0.142
**C3**	0.176	0.193	0.246	0.263
**C4**	0.205	0.236	0.250	0.263

To directly address the relationship between SoO and RoT, we compared the correlation between SoO and RoT items at the near versus far distance. Both within (r = 0.824) and beyond (r = 0.741) the perihand space, SoO and RoT showed high correlation coefficients. However, the correlation between SoO and RoT was significantly higher within the near distance than at the far distance (CI: 0.012; 0.163).

## Discussion

In the present study, we focused on the relationship between the feeling of perceiving the rubber hand as part of the body (Sense of Ownership, SoO) and that of co-localizing the sensation of the two hands (referral of touch, RoT). We did so by adopting a psychometric perspective on a large RHI data set, using mainly exploratory graph analysis methods. The literature shows contradictory results regarding the relationship between SoO and RoT. To address this issue, we first assessed the item structure and then directly compared the correlation between SoO and RoT items in different conditions.

First, we found that statements referring to SoO are central (i.e., high strength) to the overall response pattern. These statements further show a consistent correlation to the RoT statements. Second, we found indications that the RoT statements can represent its own distinct group, suggesting that despite their overall strong correlation to the ownership ratings, these statements may represent a related but separate facet of the illusion. Indeed, we found that the correlation between SoO and RoT diminished when the rubber hand is placed beyond the perihand space, suggesting that the two sensations are closely related, but respond differently to manipulations. Third, we found moderate relationships between the control and illusion statements, suggesting a careful consideration of what control items are measuring.

The RHI and related body illusion paradigms center on the concept of body ownership. They are considered to be experimental manipulations of the sense of ownership over body parts or the whole body (Ehrsson, 2011). Based on the first RHI study by Botvinick and Cohen (1998), most questionnaires deployed in these experiments use statements reflecting the experience of ownership over the artificial body, but also statements that reflect a sensation of referring the touch to the rubber hand. Our data confirm that the statements concerning these two categories form a distinct community, with the former being central to the questionnaire responses and the latter showing high correlations to the ownership responses. This affirms the importance of the ownership dimension in RHI paradigms. Previous investigations have equally shown that the experience of ownership is a key component within the experience of the RHI, accounting for one-third (Longo et al., 2008) or one-quarter (Romano et al., 2021) of the variance.

Although both SoO and RoT are the main components of the illusion, and indeed are highly correlated, we found indications for their possible separation in the bootstrap analysis. The averaged loadings show the relative contribution of each item in each community, averaging across all the bootstrap replications. Even though the solution with four communities had a low likelihood, when a fourth community was recovered, it was loaded by the RoT items. Also, the items assessing RoT had, on average, higher loadings on the fourth community than on the first one (i.e., the ownership community). This may suggest that RoT statements may indeed aggregate to form their own distinct subclass of statements, that can be separated from the ownership statements. Thus, although the relationship between SoO and RoT is solid and replicable, it may be broken in specific conditions. We further investigated which conditions may weaken the relationship between SoO and RoT by assessing the correlation between these statement categories at a close and a far distance of the rubber hand placement. Although both statement classes remained correlated at even this far distance, their correlation was significantly weaker. This suggests that under specific circumstances these two dimensions within the experience of the RHI can diminish, and that they respond differently to specific manipulations, and thus may be considered not to be equivalent. This conclusion was also supported by the EGA results. Indeed, by looking at the correlation inside the first community, we can clearly see that SoO items show stronger relationships with each other than with the RoT statements. Similarly, RoT items are more related within each other than with the ownership statements.

This is in line with previous observations that have shown that, once separated, these statement classes may be rated differently (Kalckert, Bico et al., 2019a; Reader et al., 2021). For example, Kalckert, Perera et al. (2019b) found indications that both statement classes may be differently affected by distance manipulations. Ownership ratings were rated highly within a close distance to the participant ´s hand whereas the RoT statements could be rated high even at further distances, at which ownership is typically denied. Thus, ownership may be more localized and restricted within specific perceptual boundaries. In a re-analysis of three different data sets by Reader et al. (2021), it has been shown that there is a consistent pattern emerging when inspecting RHI data this way: RoT is consistently rated higher than SoO, and some participants can seemingly affirm one without the other (e.g., a participant may affirm RoT, but not SoO). Moreover, qualitative research has equally shown that both SoO and RoT are reported as distinct experiences within the illusion (Moguillansky et al., 2013).

Our results go in the same direction, suggesting that RoT and SoO are different facets of the RHI. It has been reported that referral of touch may precede the ownership sensation and be a determinant of its manifestation (Makin et al., 2008; Reader et al., 2021). Indeed, referral of touch tends to be reported more strongly and more frequently than the feeling of ownership over the hand (Kalckert, Bico et al., 2019a; Reader et al., 2021). Previous studies also demonstrated referral of touch experiences in peripersonal space (Guterstam et al., 2016). Guterstam and colleagues (2016) showed that the integration of spatio-temporally congruent visual stimuli in the perihand space (i.e., a brush in mid-air) and tactile signals results in the multisensory perception of the rubber hand being touched by an invisible magnetic force “radiating” from the brush. The authors hypothesized that an object moving within peripersonal space represents a potential impending tactile sensation (Graziano & Cooke, 2006), which facilitates the integration of vision and touch, resembling the receptive field properties of peripersonal space neurons. Such observations and others have supported the idea that the processes underpinning the rubber hand illusion rely on peripersonal space mechanisms (see, e.g., Brozzoli et al., 2012; Serino, 2019; Zopf et al., 2010), and our results are consistent with this view.

We hypothesize that the referral of touch is more flexible than ownership, maybe because the latter is strongly linked to beliefs and experiences about the actual body and requires experiencing proprioceptive sensations (Reader et al., 2021), and the former is more related to multisensory perceptual integration. In the previous investigations of Longo et al. (2008) and Romano et al. (2021), the RoT item had a relatively low loading, inferior to the ownership items. One may argue that this indicates a relatively minor role within the illusion experience of RHI studies. Still, it should be noted that only one item (statement 8) reflects this experience of touch referral, whereas about four items (statements 1, 3, 4, and 5) can be classified as ownership-related items in these questionnaires. Similarly, a network study on a Full-Body Illusion-like paradigm found a community related to SoO (two items), but the one item assessing RoT showed no connection with the network (Tosi & Romano, 2023).

Besides these affirmative observations, we also noticed that the control statements are closely related to the items addressing the illusion. Our results confirm that the core cluster of the responses is based on the ownership ratings, but notably, the control statements show generally a positive correlation to the core illusion statements. Moreover, the variation between conditions (i.e., the delta score) was unidimensional, suggesting a tight connection between the control and illusion items. Studies have taken different approaches in the questionnaire analysis and in some instances have included the control items as part of the overall analysis approach (e.g., by subtracting the control ratings from the illusion ratings; see also Kalckert, Bico et al., 2019a). Our results give further empirical support for the common practice of comparing the illusion ratings against a control condition rating, and discourage comparing or computing illusion scores by subtracting the control items from the illusion items. The overall positive correlation of these control items may suggest that they are modulated by the illusion experience, or in other words, that their variation is consistent with the experienced facets of the illusion experience. The study by Botvinick and Cohen (1998) already indicated that some of the control statements that were predicted not to be part of the experience (e.g., statements 7, 8, and 9) show a range of ratings that suggest that some of these were affirmed by at least some participants. The process of subtracting control items from illusion items presents two distinct risks. Firstly, the lack of control for sensations unrelated to the illusion may result in the generation of spurious scores. Secondly, the elimination of some illusion-related effects may be a consequence of this approach.

The tendency to positively affirm such statements has been proposed to be explainable with the constructs of cognitive biases, hypnotic suggestibility, or demand characteristics (Lush et al., 2020). However, it has been shown that they have a very small (or even null) effect on the illusion, and subjective reports are mostly influenced by the difference between experimental and control conditions (Slater & Ehrsson, 2022). However, while the RHI is understood to be a perceptual illusion, other aspects like personality traits (Burin et al., 2019), individual differences (Romano et al., 2021), delusional ideation (Louzolo et al., 2015), schizotypy (Asai et al., 2011), and sensory suggestibility (Marotta et al., 2016) may modulate the RHI experience.

Further, we have noted that some of the control items appeared unstable, as evidenced in the replication rate (see Fig. 1c). Consequently, these items should be considered as less optimal in their role as control questions (assuming that the control items were intended to be consistently denied or immune to the experimental manipulation). The items C3 and in particular C4 showed the lowest replication rate, as compared to the control items C1 and C2. Whereas C1 and C2 seem to suggest experiences that are relatively counterintuitive, we could question whether certain experiences as described in these statements could really have been experienced. For example, owning more than one right hand can be experienced during the RHI. Given that it has been shown that it is possible to create a multiplication of limbs (i.e., having two right hands; see, e.g., Ehrsson, 2009; Fan et al., 2021), such an experience could also potentially be realized during the RHI experience, at least temporarily. Likewise, the experience of relocating the touch somewhere between the two hands may be equally plausible. In a similar manner, the proprioceptive drift demonstrates that the felt location of the hand is not fully relocated to the actual location of the rubber hand, but somewhere between the two hands (typically, around 30% of the distance between the two hands). These observations not only have practical implications for the application of the typical RHI questionnaire, but also pose interesting questions on the nature of embodiment experiences.

Taken together, our results do provide support for the usage of SoO- and RoT-related statements. These represent core dimensions within the experience of the illusion. At the same time, our results highlight some caveats. A more careful use of SoO and/or RoT statements in specific experimental contexts, as well as the use of control statements. This warrants further investigations of the psychometric properties of questionnaires used in body illusion experiments, and in the light of their results, further reflections on the nature of these experiments and the way these are interpreted.

## Data Availability

The data and analysis code are available via the Open Science Framework (OSF) at the following link: https://osf.io/3687e/?view_only=11995653f88b4a3cb0ca65d116555051.
